# HIV Knowledge and Associated Factors among Internet-Using Men Who Have Sex with Men (MSM) in South Africa and the United States

**DOI:** 10.1371/journal.pone.0032915

**Published:** 2012-03-12

**Authors:** Bradley H. Wagenaar, Patrick S. Sullivan, Rob Stephenson

**Affiliations:** 1 Department of Epidemiology, Rollins School of Public Health, Emory University, Atlanta, Georgia, United States of America; 2 Hubert Department of Global Health, Rollins School of Public Health, Emory University, Atlanta, Georgia, United States of America; Asociacion Civil Impacta Salud y Educacion, Peru

## Abstract

**Background:**

We compared factors associated with low HIV/AIDS knowledge among internet-using MSM in South Africa and the United States.

**Methods:**

1,154 MSM in the US and 439 MSM in South Africa, recruited through Facebook.com, completed an online survey using a US-validated HIV knowledge scale (HIV-KQ-18). Separate multivariable logistic regression models were built, one for the US and one for South Africa, using a dichotomized variable of scoring less than and equal to 13/18 (“low knowledge”) on the HIV-KQ-18 as outcome.

**Results:**

Median knowledge scores were 16/18 for both groups of respondents. For South African MSM, factors associated with low knowledge were: a high school education or less (adjusted odds ratio [aOR]: 2.5, 95% confidence interval [CI]: 1.4–4.6), not using condom-compatible lubrication during last anal sex with another man (aOR: 1.9, CI: 1.0–3.5), number of gay or bisexual acquaintances (aOR: 0.89, CI: 0.81–0.99), being unemployed (aOR: 2.2, CI: 1.0–4.6), and testing HIV negative (aOR: 0.30, CI: 0.16–0.59) or testing HIV positive (aOR: 0.15, CI: 0.03–0.74) compared to those never HIV tested. For US MSM, associated factors were: a high school education or less (aOR: 2.7, CI: 1.9–3.8), low pride and acceptance of homosexuality (aOR: 1.3, CI: 1.2–1.5), age 18–24 (aOR: 2.3, CI: 1.3–3.8) or age 50+ (aOR: 3.2, CI: 1.6–6.3) compared to age 25–29, Hispanic ethnicity compared to white non-Hispanic (aOR: 1.9, CI: 1.1–3.2), and testing HIV positive (aOR: 0.34, CI: 0.16–0.69) or testing HIV negative (aOR: 0.59, CI: 0.39–0.89) compared to those tested.

**Conclusions:**

Those developing programs for MSM in South Africa should weigh these data and other relevant factors, and might consider focusing education services towards MSM with limited education, less integration into gay/bisexual communities, no HIV testing history, limited use of condom-compatible lube, and the unemployed. In the United States, Hispanic MSM, those with limited education, no HIV testing history, low pride/acceptance of homosexuality, and those aged 18–24 or 50+ may be at risk for gaps in HIV knowledge.

## Introduction

Since the emergence of HIV as a global pandemic in the 1980s, men who have sex with men (MSM) have shared a disproportionately large burden of infection in many high-income countries in Western and Central Europe, Australia, and North America [1]. Due to this recognized high burden, MSM represent a large target population for resources on HIV/AIDS prevention, treatment, and research in these areas. By contrast, Africa's HIV/AIDS epidemic has long been understood primarily as a “heterosexual epidemic”, with an estimated 80% of HIV infections being tied to heterosexual transmission [2]. This focus has led to HIV/AIDS prevention efforts in Africa being targeted primarily to heterosexuals.

Recent epidemiological evidence has shown that MSM in Africa share a disproportionate burden of HIV infection [3]. Prevalence estimates of MSM in Africa range from 1–4% of the general population, but high levels of HIV infection and a high prevalence of MSM also engaging in sex with women has led MSM transmission to be linked to over 20% of all HIV cases in several countries of the Middle East, North Africa, and West Africa [4]–[6]. These data are at odds with the fact that most African countries have not dedicated any national HIV/AIDS funds to specifically target HIV/AIDS among MSM [6].

The 2009 UNAIDS report on universal access for MSM and transgender people highlights the global failure in addressing the needs of MSM regarding HIV/AIDS education, prevention, treatment, research, and care. One of the foci of this report is increasing access to HIV/AIDS prevention materials for MSM and transgender individuals [7]. Although increasing HIV/AIDS knowledge alone is not sufficient to promote sustainable behavior change, accurate knowledge of transmission and prevention of HIV is necessary if MSM are to adopt risk reduction strategies.

Globally, reporting on HIV knowledge among MSM is sparse. Only 33 out of 147 low and middle income countries (LMIC) reported knowledge data through the 2008 United Nations General Assembly Special Session (UNGASS) [8]. Only 2 of these 37 countries reporting UNGASS HIV knowledge data were in Africa, with Nigeria and Mauritius reporting that only 44% and 48% of MSM respectively could “correctly identify ways of preventing sexual transmission of HIV and could correctly reject major misconceptions about HIV transmission” [8]. Additionally, across all low and middle-income countries reporting knowledge scores, less than half of MSM held correct HIV knowledge.

Other studies from Sudan and Kenya indicate that MSM in Africa may have low knowledge regarding HIV prevention and transmission. More than half (55%) of a sample of MSM in Sudan and 35% of respondents in Mombasa, Kenya did not understand the link between anal sex and HIV infection [9]–[10]. By contrast, over 90% of samples of MSM in Malawi, Botswana, and Namibia understood that HIV can be transmitted through anal sex with a man [11]. However, of these samples, only 57%, 50%, and 85% respectively had ever received educational materials on preventing HIV transmission between men.

Data on levels and correlates of HIV/AIDS knowledge in Africa are essential to develop effective prevention and education strategies. Previous studies of HIV knowledge among MSM have focused on levels of HIV knowledge, but have not systematically examined factors associated with low knowledge. The present study aims to fill this gap by examining factors associated with low HIV/AIDS knowledge among MSM in South Africa and the United States using a validated HIV knowledge scale.

We chose to focus on South African and US MSM because: (1) the HIV-KQ-18 knowledge scale has only been previously validated and described among populations in the US, (2) South Africa has a large population of people living with HIV/AIDS, representing 25% of all those living in Sub-Saharan Africa, and (3) there is emerging evidence that MSM in South Africa share a disproportionate burden of HIV infection [1], [12]. This information will support the development and scale up of educational programs to address the specific needs of MSM, and may aid in optimizing existing prevention programs for MSM.

## Methods

1,154 internet-using MSM in the United States and 439 in South Africa were recruited through banner advertisements on Facebook.com targeted to men who stated they were interested in men on their Facebook profiles. Facebook ads were displayed in South Africa from June 1 to June 30, 2010 and in all US states from October 1 to November 30, 2010. Banner ads included a range of images, including groups of men, individual men of a range of ethnic and racial backgrounds, and rainbow-themed images. Facebook users who clicked on the banner ads were taken to an internet-based survey. To be eligible to begin the survey men had to report male-to-male sex in the past year.

The survey collected information on participant demographics (age, race, education, and employment), sexual orientation, the number of friends, colleagues, or acquaintances they felt identified as gay or bisexual, HIV testing behavior, knowledge of HIV transmission, condom and water-based lubrication use, and questions on gay identity. HIV knowledge was quantified using the brief HIV knowledge scale (HIV-KQ-18), an internally consistent and stable HIV knowledge scale shown to be appropriate for low-literacy populations [13]. Questions in the HIV-KQ-18 focus on basic HIV transmission and prevention and are summed to form an index of overall HIV knowledge (0–18) with non-responses and “don't know” coded as incorrect.

Gay identity was quantified using a modified version of the US-validated “Gay Identity Questionnaire” consisting of 20 questions summed to form an index of decreasing pride and acceptance of homosexuality as one moves from 0 to 80 [14]. Race in the US was classified into white non-Hispanic, black non-Hispanic, Hispanic, and other (including Asian/Pacific Islander, Native American/Alaska Native, Multi-Racial, and other). Race in South Africa was classified into black African, white/European/African, and other (including Asian, colored, and other). For both groups of respondents, individuals responding “don't know” for previous HIV testing were coded as not being tested and individuals who indicated receiving “indeterminate” HIV results were coded as testing HIV negative.

Statistical analyses were conducted using SAS 9.2 (Cary, NC, USA). Statistical significance was assessed using an alpha value of .05 and two-tailed tests. Individuals were excluded from analyses if they did not report being male and having had male-to-male sex in the past year (n = 15 US; n = 80 South Africa) or if no knowledge questions were answered (n = 883 US; n = 85 South Africa). Individuals excluded for not answering any knowledge questions did not have significantly different age or gay/bisexual peer network distributions using t-tests.

**Figure 1:**
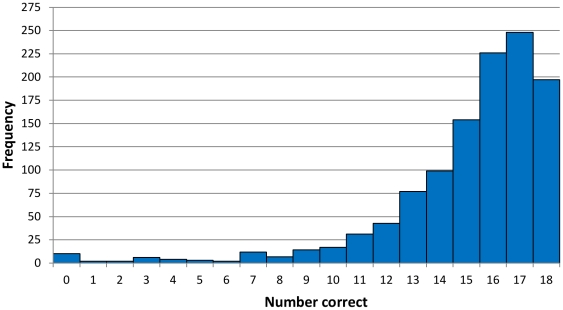
Histogram of number correct on the HIV-KQ-18 knowledge scale for 1,154 US men who have sex with men (MSM) who completed online Facebook survey, June–November 2010.

**Figure 2:**
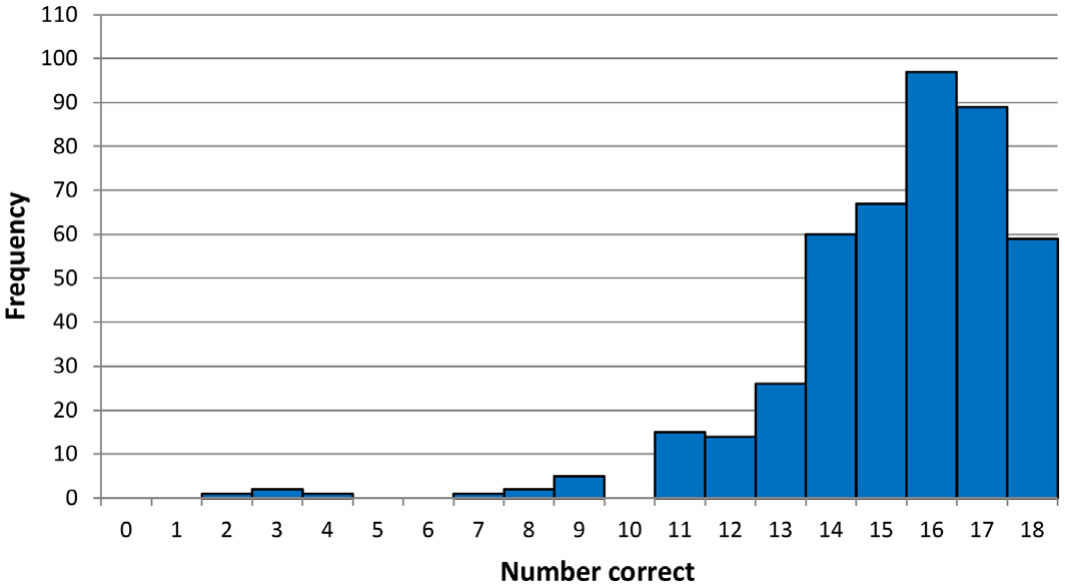
Histogram of number correct on the HIV-KQ-18 knowledge scale for 439 South African men who have sex with men (MSM) who completed online Facebook survey, June–November 2010.

Separate multivariable logistic regression models were built, one for the US and one for South Africa, using the dichotomous “low knowledge” variable as our outcome. Knowledge scores were dichotomized using a data-derived cutoff of greater than or less than and equal to 13/18 correct, with 19.9% and 15.3% scoring at this level or below in the US and South Africa respectively (Figure 1; Figure 2). We dichotomized knowledge this way because the proportional odds assumption was grossly violated for use of ordinal logistic regression with raw scores, and because there is no standard cutoff for a critical level of knowledge for the HIV-KQ-18. As a sensitivity analysis, changing this “critical knowledge” cutoff to other possible cut points of 14, 15, or 16 out of 18 correct on the knowledge scale did not meaningfully change factors associated or their magnitudes.

Variables considered for inclusion into each model were age, race, education, sexual orientation, HIV testing behavior, employment status, number of gay or bisexual friends known, having and/or had a female sex partner, relationship status (male or female), condom and lubrication use, and score on the gay identity scale. Backward selection procedures (α = .05) were used to arrive at the final models. Wald chi-square tests were used to establish significance of individual predictors, whereas likelihood ratio tests (LRT) were used to evaluate significance of groups of predictors (race, HIV-testing behavior). Hosmer and Lemeshow's goodness of fit test was used to determine if final models were adequate.

## Results

Basic demographics showed both groups of respondents to be primarily of white race, homosexual sexual orientation, and having had sex with only men (Table 1). Median knowledge scores were 16/18 correct for both cohorts, with 13.4% (59) and 17.1% (197) respondents in South Africa and the United States correctly responding to all 18 knowledge questions, respectively.

**Table 1:** Demographic and behavioral characteristics of 1,154 US and 439 South African men who have sex with men (MSM) who completed online Facebook HIV survey, June–November 2010.

Demographic or behavioral characteristic	US MSM		South African MSM	
	n (%) unless noted	IQR	n (%) unless noted	IQR
**HIV knowledge (number correct / 18)**	16 (median)	3	16 (median)	3
**Number acquaintances gay or bisexual**	20 (median)	51	20 (median)	40
**Gay identity scale**	15 (median)	23	11 (median)	16
**Age**	26 (median)	20	30 (median)	13
18–24	500 (43.3)		117 (26.7)	
25–29	183 (15.9)		96 (21.9)	
30–39	163 (14.1)		140 (31.9)	
40–49	191 (16.6)		60 (13.7)	
50+	117 (10.1)		26 (5.9)	
**Race/Ethnicity (South Africa)**				
White/European/African			400 (91.1)	
Black African			20 (4.6)	
Other			19 (4.3)	
**Race/Ethnicity (United States)**				
White non-Hispanic	557 (48.3)			
Black non-Hispanic	381 (33.0)			
Hispanic	108 (9.4)			
Other	108 (9.4)			
**Sexual Orientation**				
Homosexual/ Gay	975 (84.5)		422 (96.4)	
Bisexual	149 (12.9)		12 (2.7)	
Heterosexual	3 (0.3)		1 (0.2)	
Unsure	18 (1.6)		1 (0.2)	
Other	5 (0.4)		2 (0.5)	
Missing	4 (0.4)		1 (0.23)	
**Entire Life Had Sex With:**				
Only men	620 (53.7)		266 (60.7)	
Both men and women	534 (46.4)		172 (39.3)	
**Education**				
More than high school	901 (78.1)		186 (42.4)	
High school or less	253 (21.9)		253 (57.6)	
**HIV testing history**				
Never tested for HIV	161 (14.0)		57 (13.0)	
Positive last HIV test	133/993 (13.4)		25/382 (6.5)	
Negative / indeterminate last HIV test	860/993 (86.6)		357/382 (93.5)	
**Condom use at last sex**				
No anal sex last sex with partner	284 (24.6)		101 (23.0)	
No condom last anal sex with partner	492/870 (56.6)		173/338 (51.2)	
Used condom last anal sex with partner	378/870 (43.4)		165/338 (48.8)	
**Currently in a sexual relationship (M or F)**	511 (44.4)		258 (58.8)	
**Currently employed**	733 (63.9)		383 (88.3)	
**Condom-compatible lube use last anal sex**	861 (74.6)		349 (79.5)	

For South African MSM, less than 70% of respondents correctly answered that all pregnant women infected with HIV will not have babies born with AIDS, that a natural skin condom does not work better against HIV than a latex condom, and that a person can get HIV from oral sex (Table 2). For US MSM, less than 70% of respondents correctly answered that there is a female condom that can help decrease a woman's chance of getting HIV, and that a natural skin condom does not work better against HIV than a latex condom.

**Table 2:** 1,154 US and 439 South African men who have sex with men (MSM) who completed online Facebook survey answering HIV-KQ-18 questions correctly, incorrectly, “don't know”, or by skipping, June-November 2010.

	US MSM			South African MSM		
HIV-KQ-18 Question (correct answer)	Correct n (%)	Incorrect n (%)	Don't Know/ Skipped n (%)	Correct n (%)	Incorrect n (%)	Don't Know / Skipped n (%)
1. Coughing and sneezing DO NOT spread HIV (T)	948 (82.1)	122 (10.6)	84 (7.3)	390 (88.8)	37 (8.4)	12 (2.7)
2. A person can get HIV by sharing a glass of water with someone who has HIV (F)	1045 (90.6)	37 (3.2)	72 (6.2)	403 (91.8)	31 (7.1)	5 (1.1)
3. Pulling the penis out before a man climaxes/cums keeps his partner from getting HIV during sex (F)	1029 (89.2)	53 (4.6)	72 (6.2)	392 (89.3)	21 (4.8)	26 (5.9)
4. A woman can get HIV if she has anal sex with a man (T)	982 (85.1)	100 (8.7)	72 (6.2)	377 (85.9)	40 (9.1)	22 (5.0)
5. Showering or washing one's genitals / private parts after sex keeps a person from getting HIV (F)	1043 (90.4)	30 (2.6)	81 (7.0)	425 (96.8)	6 (1.4)	8 (1.8)
6. All pregnant women infected with HIV will have babies born with AIDS (F)	830 (71.9)	151 (13.1)	173 (15.0)	286 (65.1)	109 (24.8)	44 (10.0)
7. People who have been infected with HIV quickly show serious signs of being infected (F)	1078 (93.4)	22 (1.9)	54 (4.7)	416 (94.8)	6 (1.4)	17 (3.9)
8. There is a vaccine that can stop adults from getting HIV (F)	989 (85.7)	33 (2.9)	132 (11.4)	376 (85.6)	22 (5.0)	41 (9.3)
9. People are likely to get HIV by deep kissing / putting their tongue in their partner's mouth (F)	907 (78.6)	104 (9.0)	143 (12.4)	372 (84.7)	34 (7.7)	33 (7.5)
10. A woman cannot get HIV if she has sex during her period (F)	1004 (87.0)	34 (2.9)	116 (10.1)	416 (94.8)	5 (1.1)	18 (4.1)
11. There is a female condom that can help decrease a woman's chance of getting HIV (T)	792 (68.6)	142 (12.3)	220 (19.1)	333 (75.9)	52 (11.8)	54 (12.3)
12. A natural skin condom works better against HIV than a latex condom (F)	790 (68.5)	19 (1.6)	345 (29.9)	256 (58.3)	10 (2.3)	173 (39.4)
13. A person will NOT get HIV if they are taking antibiotics (F)	1066 (92.4)	18 (1.6)	70 (6.1)	415 (94.5)	6 (1.4)	18 (4.1)
14. Having sex with more than one partner can increase a person's chance of becoming infected with HIV (T)	1088 (94.3)	33 (2.9)	33 (2.9)	427 (97.3)	9 (2.1)	3 (0.68)
15. Taking a test for HIV one week after having sex will tell a person if she or he has HIV (F)	916 (79.4)	81 (7.0)	157 (13.6)	333 (75.9)	47 (10.7)	59 (13.4)
16. A person can get HIV by sitting in a hot tub or a swimming pool with a person who has HIV (F)	1048 (90.8)	30 (2.6)	76 (6.6)	418 (95.2)	6 (1.4)	15 (3.4)
17. A person can get HIV from oral sex (T)	880 (76.3)	162 (14.0)	112 (9.7)	301 (68.6)	79 (18.0)	59 (13.4)
18. Using Vaseline or baby oil with condoms lowers the chance of getting HIV (F)	1000 (86.7)	29 (2.5)	125 (10.8)	387 (88.2)	13 (3.0)	39 (8.9)

Hosmer and Lemeshow goodness-of-fit tests for each final model revealed no significant lack-of-fit (SA, p = .60; US, p = .95). Sexual orientation, having/and or had a female sex partner, sexual relationship status, and condom use were eliminated from both final models through backward elimination.

### Factors Associated with Low Knowledge among Both Cohorts

Controlling for all other factors in the final models, compared to MSM with greater than 12 years of education, men with less than 12 years of education were 2.5 and 2.7 times as likely to score “low” on HIV knowledge in South Africa and the US, respectively (SA, p = .003; US, p<.001; Table 3). Additionally, previous HIV testing as a group predictor was significantly associated with higher HIV knowledge scores (SA, p<.001; US, p = .006). South African MSM testing HIV positive were 85% (p = .02) less likely to score low on HIV knowledge, and men who tested HIV negative were 70% (p<.001) less likely, both compared to men never HIV tested. US MSM testing HIV positive were 66% (p = .003) less likely to score in the lowest quintile of knowledge scores, and men who tested HIV negative were 41% (p = .012) less likely, both compared to men never HIV tested.

**Table 3:** Multivariable logistic regression models for 1,154 US and 439 South African men who have sex with men (MSM) using scoring in lowest quintile on HIV-KQ-18 knowledge scores as outcome.

	US MSM	South African MSM
Covariates	aOR (95% CI)^a^	aOR (95% CI)^a^
Gay identity scale (10 point change)	1.3 (1.2–1.5)†	n.s.
Education level<12 years	2.7 (1.9–3.8)†	2.5 (1.4–4.6)*
Not employed	n.s.	2.2 (1.0–4.6)*
Did not use lube last anal sex with man	n.s.	1.9 (1.0–3.5)*
Number acquaintances gay or bisexual (10 point change)	n.s.	0.89 (0.81–0.99)*
**HIV testing history** b		
Never HIV tested	1 (reference)	1 (reference)
Tested HIV positive	0.34 (0.16–0.69)*	0.15 (0.03–0.74)*
Tested HIV negative	0.59 (0.39–0.89)*	0.30 (0.16–0.59)*
**Age group** c		
25–29	1 (reference)	
18–24	2.3 (1.3–3.8)*	
30–39	1.5 (0.77–2.8)	
40–49	1.4 (0.76–2.7)	
50+	3.2 (1.6–6.3)†	
**Racial/Ethnic group** d		
White non-Hispanic	1 (reference)	
Hispanic	1.9 (1.1–3.2)*	
Black non-Hispanic	1.5 (0.98–2.3)	
Other race	1.0 (0.56–1.9)	

* P<.05 (Wald ×^2^).

† P<.001 (Wald ×^2^).

n.s. = eliminated through backward selection for given model.

a Variables considered for inclusion into each model: age, race, education, sexual orientation, HIV testing behavior, employment status, number of gay or bisexual friends known, having and/or had a female sex partner, relationship status, condom and lubrication use, and scores on the gay identity scale. Backward selection procedures (α = .05) were conducted separately for US and South African MSM.

b Construct p-values for HIV testing history were p = .006 (US MSM) and p<.001 (South African MSM).

c Construct p-values for age group were p = .002 (US MSM) and non-significant (South African MSM).

d Construct p-values for racial/ethnic group were p = .049 (US MSM), non-significant (South African MSM).

### Factors Associated with Low Knowledge among South African MSM

First, unemployed South African MSM were 2.2 times as likely to score low on knowledge scores, compared to those who were employed (p = .042). Second, MSM not using condom-compatible lubrication during last anal sex with another man were 1.9 times as likely to score low on HIV knowledge (p = .036). Last, for each ten point increase in the number of gay or bisexual friends known, the odds of scoring low on HIV knowledge decreased 11% (p = .024).

### Factors Associated with Low Knowledge among MSM in the United States

For each ten point increase on the gay identity scale (decreasing pride and acceptance of homosexuality) the odds of scoring in the lowest quintile on knowledge scores increased by 30% for US MSM (p<.001). Age as a group predictor was significantly associated with knowledge scores (p = .002). Compared to US MSM age 25–29, those age 18–24 and 50+ were 2.3 (p = .002) and 3.2 (p<.001) times as likely to score in the lowest quintile on knowledge scores, respectively. Finally, race as a construct was significantly associated with the odds of scoring low on HIV knowledge (p = .049). Compared to white non-Hispanics, Hispanics were 1.9 times as likely to score low on HIV knowledge (p = .018).

## Discussion

Among Facebook-using MSM in the US and South Africa, HIV/AIDS knowledge levels were high: the median respondent in both groups of respondents missed only 2 out of 18 questions. In both US and South African MSM, men with less than a high school education had significantly lower HIV/AIDS knowledge. This is in corroboration with a large body of research around the world showing that as general education level increases, so does knowledge of HIV/AIDS prevention and transmission [15]–[18]. Efforts to increase general educational opportunities for MSM in South Africa and the US, would be helpful inherently, and might also support higher HIV/AIDS knowledge.

Second, previous HIV testing was associated with having higher knowledge for MSM in the US and South Africa. Although causality is unclear from our study design, we hypothesize that MSM who have tested for HIV and received negative results gain some knowledge through contact with testing facilities, while individuals testing positive show a trend towards further increased knowledge due to continued contact with healthcare providers, educational materials, or peer groups. According to our data, traditional HIV/AIDS education and counseling associated with voluntary counseling and testing centers (VCT), hospitals, or other care centers is associated with higher levels of HIV knowledge among MSM.

Among South African MSM, having fewer acquaintances gay or bisexual was associated with lower HIV/AIDS knowledge, which could indicate that, in South Africa, peer networks are key avenues where HIV/AIDS information is shared. Additionally, after accounting for HIV testing behavior, not using condom-compatible lubrication during anal sex was associated with lower HIV knowledge in South Africa. South African MSM who receive condom-compatible lubrication from a community based organization or an NGO may also receive educational materials, making use of lubrication use a marker for access to outreach or comfort with service utilization.

For US MSM, there was a trend towards lower HIV knowledge among some racial and age groups. Hispanic MSM had significantly lower HIV/AIDS knowledge and black non-Hispanic MSM showed a trend towards lower knowledge, compared to White non-Hispanic MSM. Thirty years after HIV was first propelled into global consciousness, US MSM of color are at risk for gaps in HIV knowledge at a time when new HIV infections among these men are increasing.

US MSM age 18–24 or 50+ were at significantly increased risk for low HIV knowledge compared to men age 25–29. That these two age groups have the lowest HIV knowledge is troubling because sex with older partners has been shown to be associated with HIV infection, and US MSM age 50+ have an estimated HIV prevalence of 25% compared to 10.5% for men age 18–24 [19]–[20]. Tailored educational interventions, perhaps using social media for young MSM, and other creative methods to reach older MSM are urgently needed.

A novel finding of the current study is that low pride and acceptance of homosexuality was one of the factors most strongly associated with low HIV/AIDS knowledge among US MSM. Low pride and acceptance of homosexuality may correlate with willingness or ability of US MSM to access prevention services or with the extent of peer networks through which HIV education is shared. Additional studies are needed to characterize how gay identity formation in the United States may be related to other HIV-associated factors, especially in light of the recently questioned relationship between internalized homophobia and risky sexual behaviors [21].

### Limitations

This study has several limitations. First, our findings are not generalizable to all MSM, or all Facebook-using MSM, in either the United States or South Africa. Nevertheless, according to publically available data, 58.6% (136,937,240) of over-18 individuals in the US and 8.5% (3,446,000) of over-20 South Africans have Facebook accounts [22]–[24]. Compared to the general population, our samples over-represent individuals who identify as white in South Africa (91.1% versus 9.6%) and individuals who identify as black in the United States (33% versus 12.6%) [22], [25]. This indicates that even though our sample is not representative of the population as a whole, internet-based surveys can achieve good penetration into at-risk populations in both the US and South Africa.

Second, since our Facebook advertisements were only targeted to men who explicitly state that they are “interested in” men, our sample likely consists of men who are more open with their sexuality than are MSM in general. Third, recall bias, sample selection bias, and/or social desirability bias could cause underreporting of HIV status, male sex partners, or unprotected sex and could result in misclassification. However, we judge that these biases, if they exist, are not strongly heterogeneous among those scoring high/low on HIV knowledge or between cohorts. Fourth, our choice to dichotomize the knowledge data was empirical; further research is needed to characterize epidemiologically significant critical levels of HIV/AIDS knowledge. Last, the HIV-KQ-18 knowledge scale was not developed or validated for use with MSM or with South Africans, and some of the items, such as the item about natural skin condoms, are somewhat outdated.

### Conclusions

Those developing programs for MSM in South Africa should weigh these data and other relevant information, and might consider focusing educational services towards MSM who have lower education levels, less integration into gay or bisexual communities, no HIV testing history, limited use of condom-compatible lube, and who are unemployed. In the United States, Hispanic MSM, those who have low pride and acceptance of homosexuality, those who have not tested for HIV, those with less than a high school education, and those aged 18–24 or over 50 may be at risk for gaps in HIV knowledge.

As we begin to work with recently acknowledged at risk populations, such as MSM in South Africa, or strive to strengthen service delivery in the US, programs must be tailored to populations most at risk. As further research is conducted, our study shows that online surveys are one appropriate way to reach populations of MSM in the US and South Africa.
