# Correction: HIV Knowledge and Associated Factors among Internet-Using Men Who Have Sex with Men (MSM) in South Africa and the United States

**DOI:** 10.1371/annotation/7910bc43-9371-4ad5-aa5d-900161daf089

**Published:** 2012-07-10

**Authors:** Bradley H. Wagenaar, Patrick S. Sullivan, Rob Stephenson

There is an error in the Results section of the Abstract. The correct Results statement should read: "Median knowledge scores were 16/18 for both groups of respondents. For South African MSM, factors associated with low knowledge were: a high school education or less (adjusted odds ratio [aOR]: 2.5, 95% confidence interval [CI]: 1.4–4.6), not using condom-compatible lubrication during last anal sex with another man (aOR: 1.9, CI: 1.0–3.5), number of gay or bisexual acquaintances (aOR: 0.89, CI: 0.81–0.99), being unemployed (aOR: 2.2, CI: 1.0–4.6), and testing HIV negative (aOR: 0.30, CI: 0.16–0.59) or testing HIV positive (aOR: 0.15, CI: 0.03–0.74) compared to those never HIV tested. For US MSM, associated factors were: a high school education or less (aOR: 2.7, CI: 1.9–3.8), low pride and acceptance of homosexuality (aOR: 1.3, CI: 1.2–1.5), age 18–24 (aOR: 2.3, CI: 1.3–3.8) or age 50+ (aOR: 3.2, CI: 1.6–6.3) compared to age 25–29, Hispanic ethnicity compared to white non-Hispanic (aOR: 1.9, CI: 1.1–3.2), and testing HIV positive (aOR: 0.34, CI: 0.16–0.69) or testing HIV negative (aOR: 0.59, CI: 0.39–0.89) compared to those never HIV tested." 

